# Supplementary material for: Detection and Typing of Human Enteroviruses from Clinical Samples by Entire-Capsid Next Generation Sequencing
Source: Viruses. 2021 Apr 8;13(4):641. doi: 10.3390/v13040641 (PMC8070635; doi:10.3390/v13040641)
Supplement: Supplementary file 1 [file viruses-13-00641-s001.zip › viruses-1172568-supplementary-figures.pdf]

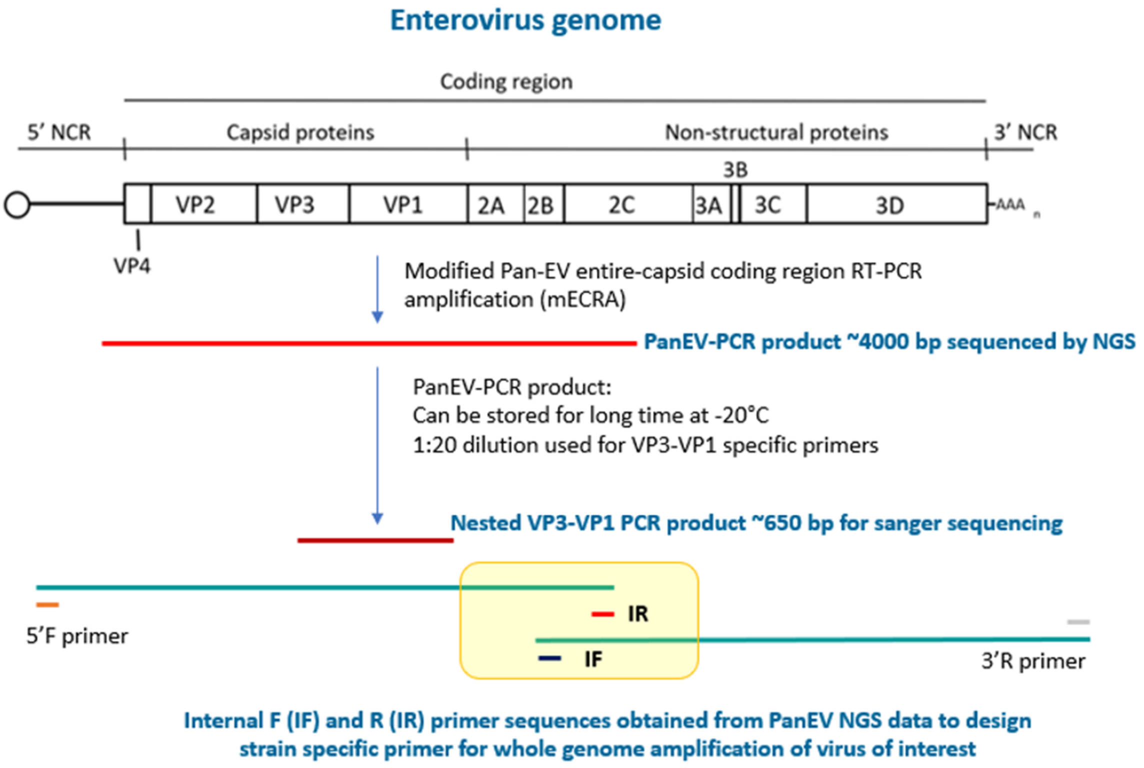

Figure S1. Generation of whole genome of virus of interst strategy described previously [12].

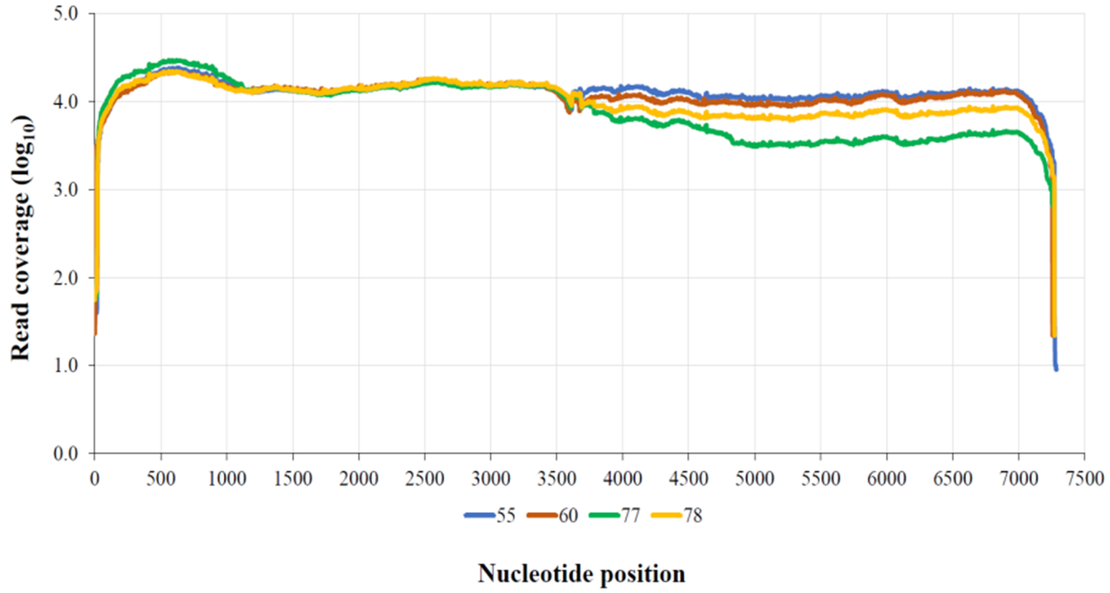

Figure S2. Genome coverage of EV-D68 strain from CLI-B3-55, 60, 77 and 78 clinical samples. Filtered reads were mapped to the final consensus sequences generated by de novo assembly. The number of sequence reads at each nucleotide position is shown.
